# Financial Support to Eligible Countries for the Switch From Trivalent to Bivalent Oral Polio Vaccine—Lessons Learned

**DOI:** 10.1093/infdis/jiw577

**Published:** 2017-07-01

**Authors:** Stephanie Shendale, Margaret Farrell, Lee M. Hampton, Jennifer B. Harris, Tasleem Kachra, Feyrouz Kurji, Manish Patel, Alejandro Ramirez Gonzalez, Simona Zipursky

**Affiliations:** 1 Expanded Programme on Immunization, World Health Organization, Geneva, Switzerland;; 2 UNICEF Programme Division, New York;; 3 Global Immunization Division, Centers for Disease Control and Prevention, Atlanta, Georgia;; 4 Bill & Melinda Gates Foundation, Seattle, and; 5 FDK Consulting LLC, Seattle, Washington;; 6 Task Force for Global Health, Atlanta, Georgia; and; 7 Polio Programme, World Health Organization, Geneva, Switzerland

**Keywords:** polio, oral polio vaccine, OPV, trivalent OPV, bivalent OPV, finance.

## Abstract

The global switch from trivalent oral polio vaccine (tOPV) to bivalent oral polio vaccine (bOPV) (“the switch”) presented an unprecedented challenge to countries. In order to mitigate the risks associated with country-level delays in implementing the switch, the Global Polio Eradication Initiative provided catalytic financial support to specific countries for operational costs unique to the switch. Between November 2015 and February 2016, a total of approximately US$19.4 million in financial support was provided to 67 countries. On average, country budgets allocated 20% to human resources, 23% to trainings and meetings, 8% to communications and advocacy, 9% to logistics, 15% to monitoring, and 5% to waste management. All 67 funded countries successfully switched from tOPV to bOPV during April–May 2016. This funding provided target countries with the necessary catalytic support to facilitate the execution of the switch on an accelerated timeline, and the mechanism offers a model for similar support to future global health efforts, such as the eventual global withdrawal of bOPV.

## BACKGROUND

In May 2012, the World Health Assembly (WHA) [1] declared the completion of poliovirus eradication to be a programmatic emergency for global public health, in response to which the Polio Eradication and Endgame Strategic Plan 2013–2018 (the “Endgame Plan”) was developed [2]. Within 1 of its 4 major objectives, the Endgame Plan called on all oral poliovirus vaccine (OPV)–using countries and territories (of which there were 155 in 2015) to withdraw the type 2 component of OPV (OPV2) in a globally coordinated manner. OPV2 withdrawal occurred through a synchronized “switch” from trivalent to bivalent OPV (tOPV to bOPV), which took place within a 2-week window during April–May 2016. Global synchronization of tOPV cessation during this limited and specific time frame helped to minimize the risk of polio outbreaks caused by type 2 circulating vaccine-derived polioviruses (cVDPV2) after the switch [3, 4, 5]. The withdrawal of OPV2 from all immunization programs around the world was a major step for polio eradication.

The May 2012 WHA resolution also highlighted the critical need to mobilize resources and develop budget scenarios for the switch. Early switch preparations revealed that the switch would pose a critical challenge to countries in the areas of planning, coordination, implementation, and financing, with significant cost implications associated with preparing for, executing, and validating the switch. Activities that specifically needed financing included additional tOPV stock inventories, training on the importance and rationale for the switch, distribution of bOPV, collection and disposal of excess tOPV stocks, and monitoring and validation of the withdrawal of tOPV from the cold chain. Existing national resource allocation and budgeting tools, such as the comprehensive multiyear plan (cMYP) costing tool, did not necessarily include or address the specific budget needs associated with the switch [6]. In addition, the target window for the switch, 17 April 17–1 May 2016, was not formally set until October 2015 when the World Health Organization (WHO) Strategic Advisory Group of Experts (SAGE) on Immunization endorsed moving forward with the switch [7]. By that time, many countries’ immunization budgets had already been approved for the first half of 2016, so funds for activities related to the switch could not easily be made available from national budgets.

Recognizing the risks associated with lack of or delays in funding for national switch activities, the Switch Implementation and Financing Sub-Groups of the Immunization Systems Management Group (IMG) of the Global Polio Eradication Initiative (GPEI) proposed a mechanism to provide financial support to countries for operationalizing their national plans within switch timelines. (The IMG is a multipartner entity responsible for the management and coordination of the GPEI’s activities in order to achieve Objective 2 of the “Endgame Plan.” Membership includes representation from the WHO, United Nations Children’s Fund [UNICEF], the US Centers for Disease Control and Prevention [CDC], the Bill and Melinda Gates Foundation, Rotary International, and the Global Alliance for Vaccines and Immunization.) This mechanism excluded costs of vaccine purchases related to the switch. In July 2015, the GPEI Strategy Committee approved a financial support envelope of US$23.7 million to support switch activities in select countries at highest risk for a cVDPV2 outbreak following the switch, as well as countries with the greatest financial need. While numerous factors at the global, regional, and country level contributed to the successful execution of the switch, one important element of this achievement was the provision of appropriate levels of financing to specific countries to meet the additional resource needs.

## DETERMINING COUNTRY ELIGIBILITY AND ESTIMATED ENVELOPES

Eligibility for financial support was based income level and the assessed risk for cVDPV2 outbreak following the switch.

### 

#### Income level

Among the 155 countries and territories that used OPV in 2015, 82 (53%) were countries classified by the World Bank as low-income (LIC) or lower-middle-income (LMIC) countries [8].

#### Risk level

IMG classified countries within 4 tiers representing the risk for a type 2 VDPV outbreak. (Tier 1 countries were wild poliovirus (WPV)–endemic countries or countries that had reported a cVDPV2 since 2000; Tier 2 countries had reported a cVDPV1/cVDPV3 since 2000 or were large/medium countries with diphtheria-tetanus-pertussis coverage of less than 80% in the past 3 years [per WHO/UNICEF National Immunization Coverage Estimates]; Tier 3 countries were those which shared a border with Tier 1 countries that reported WPV since 2003 or countries that had experienced WPV importation since 2011 [including WPV detected in environmental {sewage} samples]; Tier 4 countries were all countries that did not fit into the first 3 tiers.) The countries in Tiers 1 and 2 were deemed to be at highest risk, with Tier 3 at moderate risk. There were 49 (32%) countries falling within Tiers 1, 2, and 3.

Considering the criteria above, and in line with GPEI’s mandate to provide financial resources to poorer countries and those at higher risk, GPEI financial switch support was made available to LICs and LMICs falling within Tiers 1, 2, and 3, of which there were 43 (28%) countries in total (Appendix A). (The original list of priority countries eligible for financial support included 39 countries. However, in October 2015, following an emergence of a cVDPV2 case in Guinea, the list of countries in each risk tier was revised and the number of eligible countries was increased to 43.)

### Costing Methodology

A model was developed to estimate envelopes of financial support that would be made available for each country, based on an analysis of OPV supplementary immunization activity (SIA)–related costs in selected Tier 1, 2, and 3 countries, available SIA budgets, and cost drivers for activities such as salaries, transportation, trainings and meetings, document production, vaccine distribution and collection, and waste management (Table 1; see Appendix B for a full list of line items considered in the model). To estimate standard switch budgets as the basis for establishing funding envelopes, eligible countries were divided into 4 categories according to the following criteria: operational costs (high/low) based on 2014 and 2015 OPV SIA costs of above or below US$0.4 per child, and VDPV risk (high/low) based on the occurrence of cVDPV2 since 2000 (Table 2). The latter categorization (high/low VDPV risk) was important for estimating budgetary needs for monitoring and validating the removal of tOPV from the cold chain. The globally recommended monitoring strategy was aimed at identifying cold chain stores and health facilities holding tOPV after the switch. The assumption for budget planning was that high-risk countries would require more personnel and a higher level of coordination for monitoring activities.

**Table 1.** Main Cost Drivers Assumed in the Model

Budget Category	Average Share of Budget (%)	Main Activities Included
Human resources	43%	Switch coordinators, supervisors and switch teams Cold chain and data management
Document production	3%	Development of training materials Printing of materials
Trainings and meetings	9%	Training of medical staff Training of switch teams ICC and NCC meetings
Communication and advocacy	7%	Sensitization activities Information materials
Logistics	1%	tOPV inventories Distribution of bOPV to the districts
Transport for implementation and supervision	14%	Vehicle and motorbike rentals Fuel
Monitoring	17%^a^	Switch monitors, supervisors and coordinators Transport for monitoring (vehicle/motorbikerentals, fuel) Additional follow-up in problem areas
Waste management	1%	Return of tOPV to disposal sites Disposal
Miscellaneous	5%	…
Total	100%	…

Abbreviations: bOPV, bivalent oral polio vaccine; ICC, Interagency Coordinating Committee; NCC, National Certification Committee; tOPV, trivalent oral polio vaccine.

^a^Monitoring activities were expected to account for approximately 15% of overall costs in low-risk countries, and 18%–20% for higher-risk countries where additional monitoring was anticipated.

**Table 2.** Country Categorization for Budget Estimation

VDPV RiskOperational Cost	Low Risk for cVDPV2	High Risk for cVDPV2
Low	Bangladesh, Benin, Burkina Faso, Cambodia, Congo, Cote d’Ivoire, Egypt, Haiti, Indonesia, Laos, Liberia, Mali, Myanmar, Nepal, Papua New Guinea, Philippines, Senegal, Tajikistan, Timor-Leste	**Afghanistan**, Cameroon, Guinea, **India**, Madagascar, Niger, **Nigeria, Pakistan**
High	Burundi, Central African Republic, Eritrea, Guinea Bissau, Mauritania, Mozambique, Sierra Leone, Sudan, Uganda	**Chad**, **Democratic Republic of Congo**, **Ethiopia**, Kenya, **Somalia**, **South Sudan**, Yemen
	Low support	High support

Note: Countries in **bold** are those with a large GPEI-funded workforce.

Abbreviations: cVDPV2, type 2 circulating vaccine-derived polioviruses; GPEI, Global Polio Eradication Initiative; VDPV, vaccine-derived polioviruses.

A standard budget was then developed for each of the 4 categories of countries (low risk/low cost, low risk/high cost, high risk/low cost, high risk/high cost), using the cost drivers described above, on the basis of a hypothetical country with a population of 10 million people, 8 regions, 40 districts, and 800 health facilities, with cold chain equipment capable of storing vaccine overnight. There was an assumption that 10% of health facilities would need to be monitored in the “low risk” category and 15% of facilities would need to be monitored in the “high risk” category. Modified budgets with reduced amounts of support from GPEI were developed for the 9 countries with large numbers of GPEI-funded staff, acknowledging that teams working on polio eradication in these countries should be able to undertake much of the switch-related work, especially in terms of training health-care staff and monitoring the withdrawal of tOPV. (It was expected that countries with large polio teams would not receive additional funding for human resources, bOPV distribution costs, and information, education, and communication activities, and would receive a reduced amount for transport and fuel costs.) Country envelopes were derived from these standard budgets per 10 million population, using the United Nations’ population estimates for 2015 [9]. (For India, the Effective Vaccine Management [EVM] parameters were used to determine costing in light of the variance in the model and recent EVM data [200 facilities with cold chain per 10 million population vs 800 facilities in the model].)

Table 3 provides the average operational budget, per 10 million population, generated for each category.

**Table 3.** Operational Cost (US$)/10 Million Population

VDPV Risk Operational Cost	Low Risk for cVDPV2	High Risk for cVDPV2	Countries With Large GPEI Workforces, High Risk
Low	126316	157767	36857
High	193199	230764	55957
	Low support	High support	Reduced support

Abbreviations: cVDPV2, type 2 circulating vaccine-derived polioviruses; GPEI, Global Polio Eradication Initiative; VDPV, vaccine-derived polioviruses.

### Contingency Fund

A contingency fund was built into the overall financial support budget to provide a buffer for additional support to countries if national governments were unable to manage residual switch-related costs sufficiently, including in countries affected by unexpected disasters, emergencies, or civil disorder close to the time of the switch. This buffer also allowed for the provision of catalytic funding to countries that did not meet the initial eligibility criteria for funding but would not otherwise be in a position to fully finance the switch. The contingency fund of US$3.2 million represented 14% of the total budget.

## APPLICATIONS FOR SUPPORT

During the switch planning process, the IMG notified the governments of the 43 priority countries that they were eligible to apply for financial assistance but also emphasized that this support was meant to be supplemental in nature and would only be allocated on the basis of a well-documented national switch plan that highlighted the use of other sources (for example, national governments and local donors) to support switch activities. The estimated model allocations for countries were shared with the WHO and UNICEF regional offices but were not publicly disclosed, as these figures were generated as estimates for allocating overall resources required globally and adhering to the global envelope for financial support, not as definitive financial entitlements for each country. WHO and UNICEF country and regional offices worked closely with country governments to ensure timely completion of switch implementation plans and detailed budgets, and to address any challenges encountered or anticipated.

Application packages were received by the WHO and/or UNICEF regional offices on behalf of the IMG and screened by regional office staff before submission to the IMG for review. Complete application packages included a national switch implementation plan, including information on bOPV procurement; a complete tOPV inventory across all vaccine storage levels; and a detailed budget that outlined the operational costs of the national switch plan and identified national resources secured for the switch, any resources secured from partners and local donors, and any gaps in funding that remained. An application deadline of 13 November 2015 was set to allow ample time for review of the applications at the global level. Despite this known deadline, only 5 applications were received by that date; the majority were not submitted until December or later.

## REVIEW PROCESS AND APPROVAL

In order to review in detail each country application package, a Switch Country Financial Support Review Group (“Review Group”) was convened by the IMG, including representation from UNICEF Programme Division (PD), the CDC, the Task Force for Global Health, the Bill and Melinda Gates Foundation, the WHO Expanded Programme on Immunization (EPI) and the WHO Polio team, as well as switch focal persons from each of the WHO and UNICEF regional offices. The Review Group reviewed applications by teleconference on a rolling basis over a period of 4 months between mid-November 2015 and March 2016, during which switch plans and budgets were reviewed thoroughly to ensure that plans included details on proper switch management and funding oversight, bOPV procurement plans were feasible and underway, training plans were well outlined, and monitoring and validation frameworks were specified. Summaries of applications and recommendations for support were then shared with the IMG for ultimate approval.

The model allocations were used as guidance, but each country budget was reviewed in detail on a case-by-case basis, taking into account the financial and operational realities within each country. Participation of the WHO and UNICEF regional focal points provided the appropriate insight and background needed for each application. Budgets were carefully considered within the context of other immunization- and polio-related activities to ensure the most efficient use of resources. The Review Group also suggested improvements to the budgets where applicable, and in some cases requested resubmission of a revised budget for subsequent review.

In some cases, the Review Group recommended extending support above the initial model estimation, and in others the Review Group concluded that countries were able to finance the switch with less financial support than the model had predicted, such as when substantial national financial resources for the switch were available or when integration with inactivated poliovirus vaccine introduction activities provided efficiencies. By the end of January 2016, 40 country application had been submitted and reviewed, and a total of US$14.4 million had been awarded; this was more than US$4 million under the projected budget for those countries. (The last 3 of the original eligible country applications were not submitted until February 2016.) The majority of the contingency fund remained as well. In light of this, the GPEI agreed to allow the IMG to use the same managed process to extend financial assistance on a case-by-case basis to additional countries experiencing financial constraints related to the switch because of insufficient resources allocated by the national government or budgeting cycle timelines.

By March 2016, a total of US$19.4 million in financial support for the switch was provided to 67 countries (Appendix A). The total amount was still US$4.3 million under the initial projected budget.

## ANALYSIS OF PROCESS

Support to countries for switch implementation ranged from as little as US$4000 to as much as US$2230000, with an average of US$289109 per country supported. A breakdown in the percentage of the total national switch budgets financed with GPEI support is presented in Table 5.

**Table 5.** Percentage of Total National Switch Budgets Supported by GPEI

**<50%**	25 countries
**50%–75%**	22 countries
**76%–90%**	12 countries
**>90%**	3 countries

Note: Total national budgets were only received for 62 countries.

Abbreviation: GPEI, Global Polio Eradication Initiative.

In total, 35 (52%) of the 67 country requests were for amounts greater than the estimated envelopes generated by the model, and 32 (48%) were for amounts less than the model envelopes. Upon detailed application review, however, the Review Group recommended awarding amounts equal to the model envelope to only 11 (16%) of the 67 countries. Thirty-three (49%) countries were awarded less than the model predicted; among these countries, awards were a median of 37% lower than the model prediction (range, 1%–90%). Twenty-three (34%) countries were awarded funding higher than the amount predicted by the model, with the median award being 73% greater than the model prediction (range, 4%–875%) (Table 6).

**Table 6.** Financial Support Requested and Provided, as Compared to the Model-Generated Estimated Budgets

	Country Request vs Estimated Envelope (n=67)	GPEI Support Provided vs Estimated Envelope (n=67)	GPEI Support Provided vs Amount Requested (n=67)
Greater than	35 (52%)	23 (34%)	2 (3%)
Less than	32 (48%)	33 (49%)	38 (57%)
Equal to	…	11 (17%)	27 (40%)

Abbreviation: GPEI, Global Polio Eradication Initiative.

Three of the 4 countries where the needs were, upon application review, deemed to differ most significantly from the model allocations (875%, 719%, and 409%) were from the list of the 9 countries subject to a modified budget estimation because of their large numbers of GPEI-funded staff. Of the remaining countries with large numbers of GPEI-funded staff, 3 others received amounts greater than the model predicted (43%, 47%, 49%), 1 received an amount equal to the model allocation, and 2 requested and received awards lower than the estimated amounts (58% and 36% less). Looking at the financial assistance provided to countries in relation to the amount *requested*, GPEI provided support for less than the amount requested to 38 (57%) countries (23 of the original 43 priority countries, and 15 of the additional 24). Twenty-seven (40%) countries received support equal to the amount requested. For 2 (3%) countries, the review group actually recommended providing assistance above the country request, as it was recognized that particular aspects of the plan were underbudgeted, or extenuating circumstances raised by the WHO and UNICEF regional focal points indicated that mobilization of sufficient government funds was expected to be particularly challenging.

**Figure 1.**
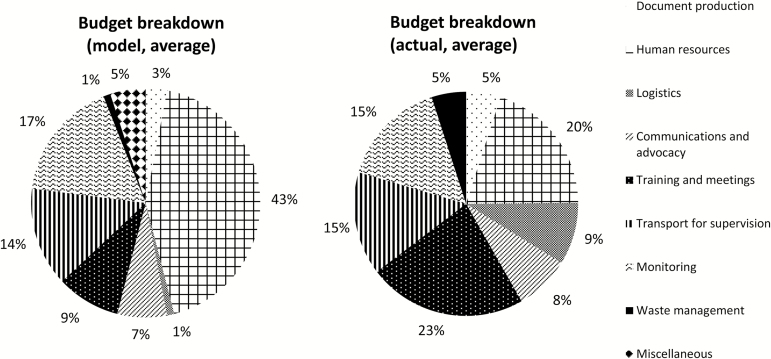
Approximate budget breakdowns, model, and actual (average, based on sample of budgets reviewed).

Budgets submitted by countries varied in format, but among the budgets reviewed, countries allocated on average 20% to human resources, 23% to trainings and meetings, 8% to communications and advocacy, 9% to logistics, 15% to monitoring, and 5% to waste management (Figure 1). Reviews considered specific activities rather than adherence to strict proportions of the total, but as a general rule, the Review Group recommended that communications activities account for no more than 10% of country budgets, and monitoring costs account for at least 10%. Certain activities were consistently excluded from GPEI funding, such as launches and ceremonies, radio and television spots, new cold chain equipment, and miscellaneous and contingency budget lines.

## CASH TRANSFER AND ACCOUNTABILITY

Endorsement of the switch plan and budget by a country’s Interagency Coordinating Committee (ICC) [10], or an equivalent body that oversaw the country’s polio elimination or overall immunization efforts, was required prior to the disbursement of any funds to ensure accountability and country ownership of the activities to be implemented. Following approval by the IMG of switch support funds for a particular country, funds were generally transferred from GPEI directly to the WHO office in that country. The WHO country office then transferred the funds to the relevant Ministry of Health or, in some cases, implemented activities directly at the request of the national government. In some cases, a portion of the funding was transferred through UNICEF PD to UNICEF country offices for activities that would be coordinated by UNICEF at the country level. Expenditures were then required to be reported through existing mechanisms to the WHO country offices within 3 months of implementation. Only 4 (6%) of the supported countries reported difficulty with implementing some switch-related activities, or last minute revisions to implementation plans because of funding problems. Ultimately, all countries were able to successfully complete the switch.

## DISCUSSION

In total, support was awarded to an additional 24 countries beyond the 43 included in the initial budgeting exercise, and the overall expenditure still resulted in a US$4.3 million savings to the program compared to the original global switch budget. The model estimated a need of US$23.7 million for the original 43 priority countries, but in practice, only US$16.6 million was needed to support those countries. The overestimation of the amount of financial support needed by those countries deemed of highest concern for the switch allowed support to be extended to additional countries, while still remaining within the global switch budget.

This overestimation highlights the limitations of using a model to estimate country needs, especially when the availability of funds at national level is not accounted for in the model. It also highlights the importance of close review and follow-up on country budgets, and strong advocacy for national financial commitment to the switch. Nevertheless, the model provided a basis for estimating countries’ funding needs in the context of the unprecedented global switch from tOPV to bOPV. Overall, the combination of the initial budget estimates from the model and a flexible mechanism for adjusting the funds disbursed based on the countries’ applications allowed GPEI to support countries to successfully achieve the endgame timelines.

Due to the short lead-time for the switch, the model was developed rapidly and may not have considered all the relevant costs for the switch. Compared to actual budgets received, the model underestimated the proportion of switch costs that would be allocated to training and logistics, and overestimated the share needed for human resources. If a model is to be used again for future withdrawals, data from actual switch budgets should inform these parameters. However, not all countries used the standard budget template, which limits the ability to make direct comparisons between the model and actual budget line items and to analyze trends across countries.

Resources were provided to countries on the basis of their actual budgets after vetting by the WHO and UNICEF regional offices and the Review Group, and the amounts awarded to individual countries sometimes differed considerably from model predictions. The greatest deviations were seen with 3 of the countries subject to modified budget estimations because they had large numbers of GPEI funded staff. This shows that the expectation for reduced support could not be uniformly applied to all countries with large numbers of GPEI-funded staff, and that a more targeted approach was needed.

By all accounts, the financial support mechanism was a critical element to the smooth execution of the switch. In many countries, the financial application process with its requirement of detailed switch plans and budgets, as well as the hands-on approach by regional focal points, was instrumental in initiating country planning processes and national resource mobilization. It also helped to ensure that no national government refused or was unable to participate in the switch because of a lack of financial resources.

Only 4 (6%) of the 67 supported countries reported failure to implement some switch-related activities because of a lack of funding. Follow-up investigation revealed instances where funding was delayed in reaching lower levels as a result of delayed requests for disbursement from WHO offices or bureaucratic backlogs within a country’s government. Earlier disbursement of funds to WHO country offices may have facilitated countries’ timely access to funds. Although limited, there were a few instances where it became clear after the review and approval of funding that the government had not, in fact, agreed to contribute the portion of financial support as indicated in the application. In 1 case, a supplementary application was filed and additional funds were disbursed to the country. In other cases, the switch budgets were trimmed. The requirement for ICC endorsement of the plans and budgets before disbursing funds was an attempt to ensure that the financial contribution attributed to the national governments had indeed been confirmed. The fact that some governments did not contribute the expected amount highlights the importance of confirming the national contributions to similar efforts earlier in the review process in the future.

To manage application review, approval, and disbursement timelines more efficiently and effectively from the global level, the funding process should be initiated even earlier before future OPV withdrawals, with the preparation of solid country plans and budgets being a prerequisite to reviewing applications for assistance. Despite a deadline of mid-November 2015, over half of the applications were not submitted until December or later; thus, setting earlier and stricter deadlines in the future may facilitate a more timely planning process. Confirmation of the withdrawal dates as far in advance as possible (ideally, at least 12–18 months) should allow governments to account for these activities in their national budgets and EPI work plans. Not only should this reduce countries’ reliance on external financial assistance by allowing for adequate forecasting in national budgets, but also provide a clearer picture on financial gaps remaining. Funding application deadlines should be set a minimum of 6 months before the date that funds are needed to arrive in the country to allow sufficient time for review and disbursement.

Unique roadblocks, such as the closure of WHO’s financial biennium, hindered efforts to disburse funds to countries in late 2015 for use in early 2016. If these events cannot be avoided entirely in the next phase of OPV withdrawal, they should be accounted for early in the planning process. Similarly, any restrictions unique to specific countries (eg, sanctions or political barriers) that may prevent funding from certain donor sources should be anticipated so that alternative sources of funding can be identified if needed.

Finally, early consultation with the WHO and UNICEF regional offices regarding management of funds at the country level (ie, by WHO and/or UNICEF country offices and channels for disbursement) is imperative to the smooth transfer of funding, and decisions on this matter should take into consideration the administrative process, program support costs, and lead times needed at each respective organization. Lessons learned from this process should allow the provision of financial support for future synchronized global immunization activities, such as bOPV withdrawal, to be carried out even more effectively and efficiently.

**Table 4.** Summary Table of Switch Financial Support and Review Process

Total GPEI funding envelope available	US$23715722
Total GPEI funding provided	US$19370288
Eligibility criteria	GPEI financial switch support was made available to all LICs and LMICs falling within Tiers 1, 2, and 3 (based on risk of a type 2 polio outbreak following the switch).
Countries eligible for support	43
Additional countries supported	24
Time frame for application and review	Deadline for applications was 13 November 2015; however, applications continued to arrive until mid-December. First round of reviews took place between mid-November and mid-January. Additional country applications were then received, and reviews continued until mid-March.
Application requirements	Countries were required to submit: 1) Copy of the National Switch Plan, in English, Spanish or French, with the national switch day scheduled *within the switch window 17 April–1 May 2016.* Plans with the switch day outside this period would not be considered for review. 2) Copy of the first tOPV inventory, or rationale for why this had not yet been done and a concrete plan and timeline for completing it. 3) A budget outlining the operational costs of the switch plan, including: • national resources from MOH secured for the switch • resources secured from partners for the switch • any remaining gaps 4) Endorsement of the switch plan by the country ICC.* * ICC endorsement was not required by the time of the application; however, it was needed to be obtained prior to disbursement of any funds.
Review process	1) Complete application packages were received and vetted by the WHO and UNICEF regional offices. 2) Applications were submitted, along with switch plan summaries, to the Review Group coordinator for circulation. 3) Review Group, together with relevant WHO and UNICEF regional focal points, reviewed applications by teleconference on a rolling basis between November 2015 and March 2016. 4) Summaries of applications and recommendations for support were shared with IMG representatives for ultimate approval, within a 1-week turnaround. 5) Funds were disbursed to WHO country offices (or in some instances, to UNICEF country offices, via UNICEF Program Division).

Abbreviations: GPEI, Global Polio Eradication Initiative; ICC, Interagency Coordinating Committee; IMG, Immunization Systems Management Group; LICs, low-income countries; LMICs, lower-middle-income countries; MOH, Ministry of Health; Review Group, Switch Country Financial Support Review Group; tOPV, trivalent oral polio vaccine; UNICEF, United Nations Children’s Fund; WHO, World Health Organization.

## Notes

***Financial support.*** This work was supported by their affiliated organizations (S. S., L. H., J. H., T. K., A. R. G., and S. Z.) or through a grant from the Bill & Melinda Gates Foundation (M. F. and M. P., grant OPP1095024); F.K. has consulted to the Bill & Melinda Gates Foundation.

***Supplement sponsorship***. This work is part of a supplement coordinated by the Task Force for Global Health with funding provided by The Bill and Melinda Gates Foundation and the Centers for Disease Control and Prevention.

***Potential conflicts of interest.*** All authors: No reported conflicts.

## Appendix A. List of Countries That Received Catalytic Funding Support for the Switch

**Table:**

Original List of Eligible Countries	Additional Countries Supported
Afghanistan	China
Bangladesh	Cook Islands
Benin	Dominican Republic
Burkina Faso	Democratic People’s Republic of Korea
Burundi	Gabon
Cambodia	Gambia
Cameroon	Ghana
Central African Republic (the)	Honduras
Chad	Jordan
Congo (the)	Kiribati
Cote d’Ivoire	Libya
Democratic Republic of Congo	Malawi
Egypt	Maldives
Eritrea	Mongolia
Ethiopia	Nauru
Guinea	Nicaragua
Guinea-Bissau	Samoa
Haiti	Sao Tome and Principe
India	Tanzania (United Republic of)
Indonesia	Togo
Kenya	Tonga
Lao PDR	Tunisia
Liberia	Vanuatu
Madagascar	Zimbabwe
Mali	…
Mauritania	…
Mozambique	…
Myanmar	…
Nepal	…
Niger (the)	…
Nigeria	…
Pakistan	…
Papua New Guinea	…
Philippines (the)	…
Senegal	…
Sierra Leone	…
Somalia	…
South Sudan	…
Sudan (the)	…
Tajikistan	…
Timor-Leste	…
Uganda	…
Yemen	…

## Appendix B. Line Listing of Activities Factored into the Model Budget Estimations

**Table:**

No.	Budget Category	Activity	Average Share of Total Budget
**1**	**Document production**		**3%**
1.1		Development of training materials	
1.2		Printing of training and monitoring materials	
1.3		Printing of information materials	
**2**	**Human resources**		**43%**
2.1		Switch Support Team coordinator	
2.2		Switch Support Team at regional level	
2.3		Cold chain assistants	
2.4		Data manager	
2.5		Regional supervision MOH	
2.6		District supervision MOH	
2.7		National MOH supervisor	
2.8		National MOH supervisor, driver	
**3**	**Logistics**		**1%**
3.1		tOPV inventories	
3.2		Distribution of bOPV to districts	
**4**	**Planning and preparations**		**0%**
4.1		Plan written with support of national SST	
**5**	**Communications and advocacy**		**7%**
5.1		Information meeting at national and regional levels	
5.2		Information meeting with all service providers at district level	
5.3		Meetings with special groups, private sector	
**6**	**Trainings and meetings**		**9%**
6.1		ICC meetings	
6.2		NCC meetings	
6.3		Switch Support Team national level training	
6.4		Switch Support Team regional training	
6.5		Training medical staff	
**7**	**Transport for implementation and supervision**		**14%**
7.1		Switch Support Team coordinator, fuel	
7.2		Switch Support Team coordinator, rented car	
7.3		Switch Support Team, regional, motorbike, fuel	
7.4		Regional supervision, own car, fuel	
7.5		District supervision, own motorbike, fuel	
7.6		National supervision, rented car	
7.7		National supervisor, fuel	
**8**	**Monitoring** ^**a**^		**17%**
8.1		Switch Monitoring national coordinator	
8.2		Switch Monitoring regional supervisors	
8.3		Switch Monitors	
8.4		Training Switch Monitors	
8.5		Switch Monitoring national coordinator, rented car	
8.6		Switch Monitoring national coordinator, fuel	
8.7		Switch Monitoring regional supervisor, fuel	
8.8		Switch Monitoring regional supervisor, rented motorbike	
8.9		Switch Monitors, fuel	
8.10		Switch Monitors, rented motorbikes	
8.11		Additional monitoring expenses for follow-up of problem areas	
**9**	**Waste management**		**1%**
9.1		Selection of disposal sites	
9.2		Disposal sites preparation	
9.3		Return of tOPV to the disposal site	
9.4		Disposal	
**10**	**Miscellaneous costs**		**5%**

Abbreviations: bOPV, bivalent oral polio vaccine; ICC, Interagency Coordinating Committee; MOH, Ministry of Health; NCC, National Certification Committee; tOPV, trivalent oral polio vaccine.

^a^Monitoring costs were estimated to account for 15% in low-risk countries and 18%–20% in higher-risk countries where additional monitoring was anticipated.
